# The silent losers of Germany’s export surpluses. How current account imbalances are exacerbated by the misrepresentation of their domestic costs

**DOI:** 10.1057/s41295-022-00291-8

**Published:** 2022-04-13

**Authors:** Palma Polyak

**Affiliations:** Faculty of Political and Social Sciences, Scuola Normale Superiore, Florence, Italy

**Keywords:** Global imbalances, Media frames, Distributional conflict, Germany

## Abstract

Germany’s excessive current account surpluses mirror domestic problems. They are rooted in inequality and a weak home market, creating an overdependence on exports. Why, then, are policymakers so reluctant to reduce them? This paper argues that a contributing factor is the public misrepresentation of surpluses’ domestic costs. Imbalances are narrated as distributional conflicts *between* countries, not *within* them; and bilateral trade is framed as a competition, where surplus countries win. The analysis reconstructs stakeholders’ positions and discursive strategies through media narratives and Bundestag debates, using an original dataset of public statements. It finds evidence for a systematic bias disregarding the domestic losers of surpluses. Whenever imbalances are discussed, the triggering event is outside criticism, mainly from the European Commission and the US. The ensuing debate follows an ‘us versus them’ logic, where foreign critics clash with domestic defenders—mainly the government and export-sector organisations. The success narrative and identitarian discourse about an ‘export nation’ limits left-wing actors’ room to move beyond incremental criticism. The analysis finds an effect of European integration exacerbating imbalances. Germans fend off critics by an arena-shifting strategy: pointing out that exchange rates and trade are European-level prerogatives, disregarding internal policy levers for rebalancing.

## Introduction

Compared to noisy foreign critics of Germany’s record-large current account surpluses, domestic losers of the country’s longstanding export-reliant model are less frequently heard—even though consistently spending less than what is produced is bad for Germany as well (Jacoby 2020; Klein and Pettis 2020). As closer empirical investigations reveal, ballooning trade imbalances (a growing difference between exports and imports) were not driven by superior export performance, but chronically weak domestic spending (Behringer et al. 2020a; Dao 2020; Tilford 2015). Simply speaking, Germany’s problem is not an *export surplus* but an *import deficit*. Imbalances mirror rampant inequality, anemic household consumption, a large public and private investment gap—distortions that could trigger domestic pushback. So why don’t these grievances translate to rebalancing?

An established answer of the political economy literature focuses on special interests of Germany’s powerful export sector, wary of negative cost-competitiveness effects of inflationary spending, hence blocking rebalancing (e.g., Hall 2012; Iversen and Soskice 2012). But even if exporters are ‘winning’ from suppressed spending through real depreciation (an assumption that is not a given), there are plenty of harmful side-effects, even for them. Chronic underinvestment in physical or digital infrastructure harms long-term (non-price) competitiveness. International pressure should also be an incentive to course-correct, as trade uncertainties damage business prospects. Tellingly, representatives of the export lobby have issued multiple public calls for more fiscal spending.^1^ Entrenched export interests alone do not offer a full explanation. Tapping into the literature on the discursive construction of interests (e.g. Hay and Rosamond 2002), this paper argues that the fallacy of mistaking weak imports for strong exports distorts the domestic politics of imbalances. Narrating the debate as a noisy rivalry *between* nations obscures the costs of surpluses *within* Germany.

Using an original database of stakeholders’ public statements, the investigation finds evidence for a systematic underrepresentation of domestic losers of surpluses. This may help explain why the costs of imbalances do not translate to a political program towards correcting them, not even by left-wing actors (Bremer and McDaniel 2020; Bremer 2020). The analysis of news media narratives and Bundestag debates finds that the triggering event for discussing the issue is almost exclusively outside criticism, creating an ‘us versus them’ dynamic where pressure ‘on Germany’ comes from abroad, activating domestic defenders. Surpluses are narrated in an identitarian discourse, where ‘we are an export nation’ or ‘export world champions’ are recurring tropes. Criticising the surplus amounts to criticising a source of national pride, limiting left-wing actors’ room to move beyond incremental criticism. As a further contribution, the analysis points to the impact of European integration exacerbating the political problem, enabling an arena-shifting strategy for Germany.

## Distributional conflicts generated by German CA surpluses

To establish the starting claim—excessive surpluses produce domestic losers in Germany—this section gives a short review of the empirical literature and some macroeconomic insights.

CA surpluses emerge through two main channels, *expenditure switching*, and *expenditure changing*. The first one implies a fall in the relative price of domestic goods, *shifting* spending away from imports—and boosting exports by making them more competitive (i.e. cheaper). The second one implies a fall in the *overall* level of spending, depressing imports as well as demand for domestically produced goods. Just by looking at the CA balance, it cannot be established which channel is at play, they are observationally equivalent—and this confounds distributional politics: while in the first case, surpluses yield obvious benefits through an expanding export sector, in the latter, surpluses indicate domestic costs like lower living standards or underinvestment. A further important insight is that policy interventions usually work through *both* channels. Real devaluation policies (e.g. cutting wages or public expenditure) are meant to stimulate exports by making the real exchange rate more competitive, but they simultaneously suppress the overall level of spending.

**Fig. 1:**
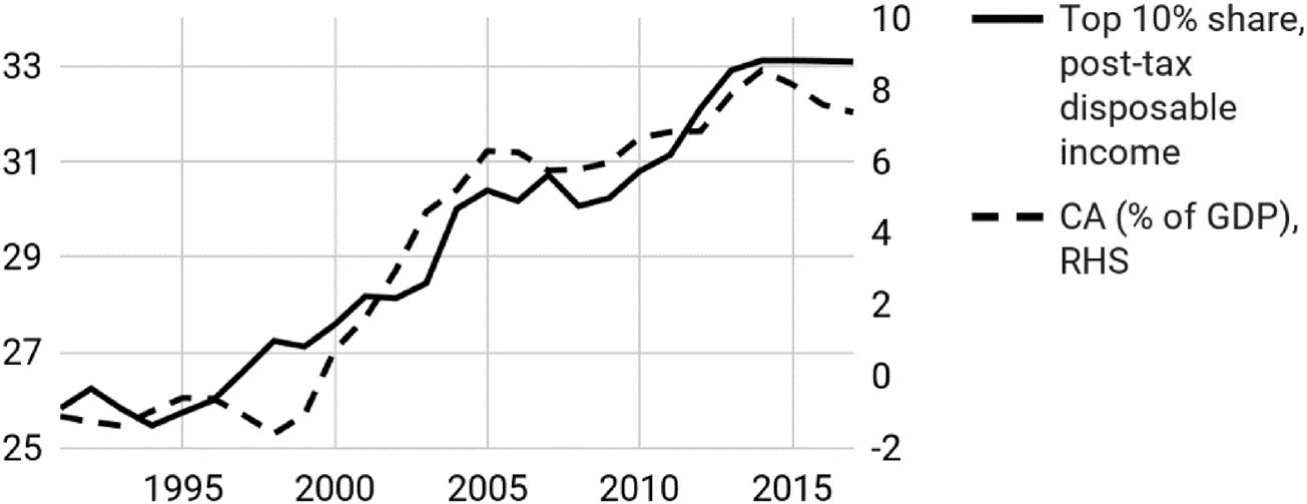
Germany’s top 10 income share and CA surplus. *Source of data* World Inequality Database, OECD

Recent empirical findings indicate that Germany’s CA imbalances are closely linked to social inequality—a strong signal for the *expenditure changing* (or import deficit) perspective. Dao (2020) finds a remarkably strong relationship between the rise of the top decile’s income share from 2000 onwards, and the surge of imbalances (the correlation coefficient is 0.94) (Fig. 1). German income inequality has been steadily increasing in the past two decades (Fratzscher 2016; Odendahl 2017; IMF 2017), poverty levels rose in parallel with a decrease in unemployment, as employee share in low-wage sectors grew. There has been a steady erosion of purchasing power in lower deciles (IMF 2019). Concentrated corporate ownership and the globalisation-induced surge of profits increased the income gap (Dao 2020).

How does inequality drive CA imbalances? A CA surplus means that residents of a country choose to spend (consume or domestically invest) less than what they produce in a given period. The residual income is ‘excess’ saving (over domestic investment; CA = *S* − *I*) that is lent abroad. These spending and saving patterns, in turn, can be driven by income redistribution.

If wages grow slower than productivity (which has been the case in Germany in the last decades), income is shifted from workers to firm-owners (Pettis 2013). Since workers have a higher marginal propensity to consume (MPC), a larger share of the overall income will be saved.

**Fig. 2:**
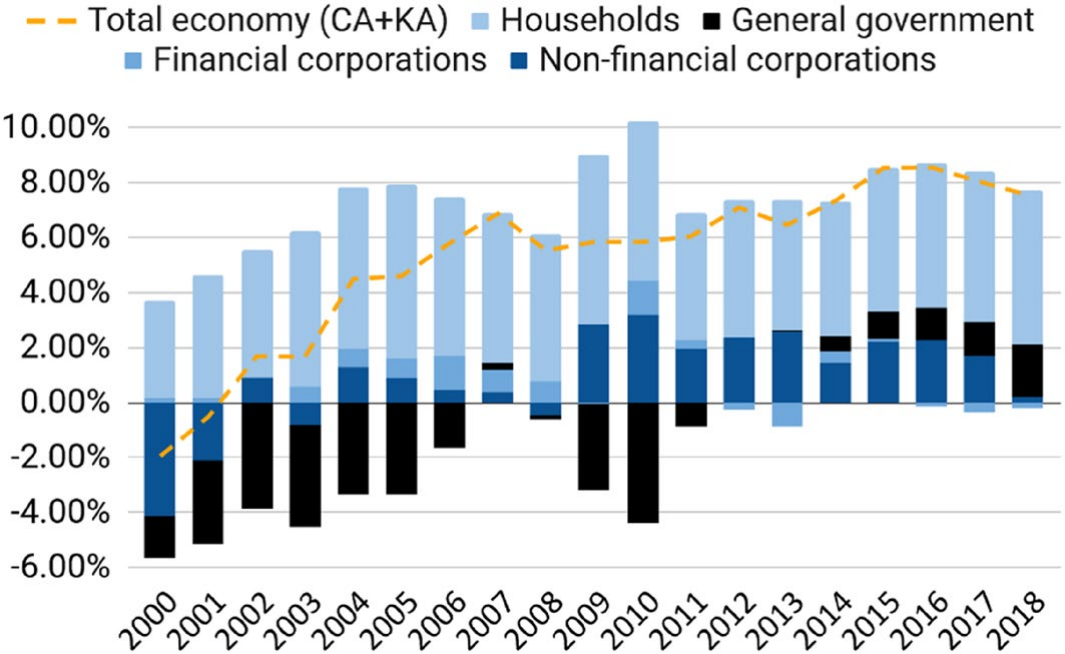
Germany, net lending by sector (percent of GDP). *Source of data* Eurostat

Decomposing the CA surplus by domestic sectors (Fig. 2) supports this narrative. On the one hand, it shows that German households save much (15% is among the Eurozone’s highest rates)—in part because of pressing demographic changes. But household savings show a constant pattern. The surplus was mostly driven by a surge in corporate savings, a sector that is generally a net investor. Non-financial corporations’ ballooning positive balance here is a proxy for retained profits (not paid as dividends) over the part that is domestically invested (Behringer et al. 2020b), underlining the growing problem of corporations ‘stashing wealth’ (Redeker 2021). Rather than counteracting the investment shortage, the government also increased savings through balanced budget policies (colloquially known as ‘Schwarze Null’ or black zero) and the constitutional debt brake (Haffert 2016).

Weak domestic spending normally undermines employment—but favorable demand shocks from China and the US boosted German job creation (Polyak 2021). But social costs beyond unemployment are severe: inequality grew, and workers in lower-income brackets saw their purchasing power drop. Underinvestment (public and private) leads to crumbling bridges and faulty digital networks, damaging long-term competitiveness in the era of rapid technological change (Roth and Wolff 2018). Germany’s public investment gap is striking—spending net of depreciation has been consistently negative since 1988 (Klein and Pettis 2020, p. 168). While unmet domestic investment needs were plenty, German savings flew into (risky) foreign assets, often beyond productive investment opportunities (Jones 2021). German investors saw a whopping 7% valuation loss on their foreign assets since 1999 (Klein and Pettis 2020, p. 86). It is an important rebuttal to authorities’ frequent reference to an aging society’s imperative to save.

In the context of globally constrained demand and near-zero interest rates (or a liquidity trap), an increase in desired saving in a surplus country leads to lower output in its trading partner (Blanchard and Milesi-Ferretti 2012). If prices (interest rates) cannot equilibrate the increased saving desire, quantities (output) become the adjustment variable. In simple terms, surplus countries are exporting *unemployment* to trading partners. It is a beggar-thy-neighbor strategy to rely on (already scarce) foreign demand without having to undertake domestic stimulus. Productive capacities of exporters continue to be utilised while deficit countries need to endure high unemployment and painful austerity to adjust or see their debt rise (Mian et al. 2020).

Table 1 sums up the costs and benefits of German surpluses, for domestic residents and for trading partners.

**Table 1:** Costs and benefits of policies driving excessive surpluses

	Germany	Trading partners
Costs	Growing inequality, lower living standards Weak demand in domestically oriented sectors Inadequate investment hurts supply potential and public goods provision Risk of trade disputes with partners Losses realised on risky foreign investments	Beggar-thy-neighbor effects of capturing already scarce demand (unemployment, slower growth) Unsustainable debt build-up
Benefits	Economic and employment growth in outward-oriented sectors Savings surplus justified by demographic trends Low indebtedness	High import-share of German exports Room for debt-fuelled consumption and growth

## The political economy of weak domestic spending

Engaging with the central puzzle (the lack of rebalancing in the face of severe domestic costs), the Varieties of Capitalism tradition (Hall 2012, 2014) stresses the role of sectoral interests. Iversen and Soskice (2012) explain how advanced nation-states are preoccupied with the policy preferences of their respective highest value-added sectors—export-oriented manufacturing in Germany, Japan, or China; and high-risk, innovative financial services in the US or UK. Even if a significant strand of German society loses out from suppressed spending, the overly powerful exporting industry benefits and blocks rebalancing. The immediate driver can be lobbying or policymakers attuned to the interests of the sector providing well-paid jobs, innovation, and human capital investment (Culpepper 2015).

If we dissect the ‘sectoral interest’ concept, the explanation leaves question marks. First, how exactly do German exporters benefit from suppressed spending? For most comparative political economy accounts (including the growth model framework pioneered by Baccaro and Pontusson 2016), the causal channel is *cost-competitiveness* through real effective exchange rates (REER): industry aims to keep spending low to prevent REER appreciation that would hurt export sales.

There are reasons to question suppressed domestic spending as the key driver of export growth. Many argue that Germany’s export success from the mid-2000s onwards was not driven by real devaluation, but links to fast-growing trading partners—suggesting export products are less sensitive to relative prices, and more to foreign activity (e.g. Danninger and Joutz 2007; Neumann 2020; Storm and Naastepad 2015). Soyres et al. (2018) show a general decline in exports’ price elasticity driven by global value chains. The debate is not settled: others estimate high price elasticities for German exports (e.g. Baccaro and Benassi 2017; Baccaro and Tober 2021). But even with an active REER channel, at least part of the effect (that is often automatically attributed to competitiveness) may be accounted for by external demand shocks, questioning the extent to which exporter firms ‘win’ from weak spending (Polyak 2021).

To assess German exporters’ interest in suppressed demand, it is also useful to disentangle *whose* demand are we talking about. Boosting government expenditure, household consumption, or investments (*G*, *C*, *I*) could all be possible avenues to rebalance the CA. Starting with fiscal rebalancing (*G*), sectoral interest-based explanations suggest that exporters are wary of expansionary fiscal policies (*G*) because they are inflationary. However, surveying stakeholders’ policy preferences in Germany, Redeker and Walter (2020) find that export-sector employers are actually quite open to fiscal rebalancing when contrasted to other alternatives. But even if real appreciation does produce costs, more spending on public goods like infrastructure also has *benefits* for business prospects. Turning to household consumption (*C*), exporters do oppose wage-based rebalancing options (e.g., minimum wage legislation). However, it is important why they might oppose. One reason—fitting the competitiveness narrative—could be that they fear it would hurt export sales through cost-competitiveness losses. But in this case, we would expect them to be similarly hostile towards other inflationary stimuli. More plausible is that wage-based rebalancing would boost labor’s share of income and decrease their profits. Finally, rebalancing could happen through government policies encouraging investment (*I*), e.g., through tax incentives or regulatory changes. If the abovementioned real appreciation channel is weak(er), these would clearly be beneficial to companies: beyond stimulating demand, they would also enhance supply capacity.

Moreover, with its degree of trade openness, the German economy, particularly the export sector depends greatly on the achievements of European market integration and a globally open trade regime. Threats of disintegration (Walter 2020) or trade conflict may further increase exporters’ stake in correcting imbalances.

To sum up, Germany’s export industry is unlikely to be the force blocking rebalancing efforts. Why, then, are policymakers so reluctant to change course? Walter et al. (2021) argue that adjustment efforts were blocked by disagreement over the specific interventions: corporate tax cuts were unacceptable for trade unions; wage increases were unacceptable for employers’ organisations. In the realms of Germany’s consensus-oriented political institutions with many institutional checks (Haffert 2016), including a rigid federal structure (Bremer et al. 2021), this gridlock is a significant force to retain the status quo. An important factor missing from this narrative is that the status quo is an attractive choice (or rather non-choice) for German policymakers as long as they can rely on external demand as an external option (Polyak 2019, 2021). Angela Merkel’s governments overrode the gridlock when facing widespread collapses of export markets, both after the global financial crisis (Schelkle 2012), and the COVID-19 shock (Sandbu 2020). Positive demand shocks from China and the US consistently masked domestic employment costs of surpluses, blunting domestic opposition against wage restraint and fiscal restraint.

However, neither of these enabling conditions can fully explain why the domestic losers of weak domestic spending and their representatives like trade unions and left-wing parties are so ineffective in advancing their grievances and pushing for a change in policy course. Low domestic unemployment supported by external demand may be a *necessary* condition, but not a *sufficient* one—rising inequality, in-job poverty, or underinvestment could all ignite political pushback.

## Theorising a disconnect between actual and perceived costs of imbalances

A more fitting theoretical approach distinguishes between actual and *perceived* distributional costs to explain the lack of German rebalancing. Tapping into the rich ideational strand of the political economy literature (Blyth 2013; Hay and Rosamond 2002; Matthijs and McNamara 2015; Matthijs 2016), distributional tensions are conceptualised as emerging from an interplay of objective reality and discursive construction. Media framing is a powerful tool in this construction process (e.g. Kneafsey and Regan 2020; Ferrara et al. 2021). Discursive framing by media narratives and problem articulation by representatives help us reconstruct distributional tensions and winner–loser relations that do not necessarily coincide with the analysis above.

Economic phenomena are framed in moral terms—narrated as stories about the good (frugal, competitive) and bad (profligate, slacker) (Matthijs and McNamara 2015). A common parallel likens firms competing on the market to economies competing against each other. Exports are often framed as a superior form of generating national income while consumption is scolded as profligate, oddly implying that all countries should rely on exports. A positive trade balance is framed as winning, and deficits are losing, even though the opposite can be argued just as (or even more) convincingly—as “surpluses are a sign that consumers are working to produce things they can’t actually use themselves. (…) ‘giving away’ goods and services in exchange for paper promises that any good dealmaker could renegotiate in the future” (Klein 2017). Similarly misleading is the microeconomic parallel between spending and saving choices of households and state budgets. This is what John Maynard Keynes famously called the *paradox of thrift*: since one’s spending is another one’s income, if all want to save at the same time, no one can save, since there is no income to save from. Hence, individually rational behavior leads to collectively damaging outcomes.

Excessive imbalances become profoundly counterintuitive for the German audience if framed as ‘too competitive firms.’ Consequently, the role of ideas has a strong case to explain why governments and voters opt for certain policies even against their economic self-interest. The analysis looks for evidence to support the hypothesised misrepresentation of domestic costs—whether imbalances are framed as a conflict *between* countries, not *within* them. The empirical marker of such a framing would be an underrepresentation of domestic stakeholders’ grievances in the debate.

Such a portrayal of imbalances can be reinforced by a debate that revolves around traditional levers of trade policy: external variables like exchange rates and tariffs as opposed to policies boosting domestic demand. As explained above, these channels are called *expenditure switching* and *expenditure changing*. The second hypothesised pattern is that the data will reveal the dominance of frames related to competitiveness or expenditure switching.

If the debate remains focused on expenditure switching—nominal euro exchange rates or tariffs—a convenient ‘arena-shifting’ strategy (Flinders and Buller 2006) opens for the German side: both monetary policy and trade policy are delegated to the European level, so German policymakers can stress their limited policy discretion. The analysis is expected to find evidence for this strategy.

## Data and methods

The empirical strategy is the qualitative content analysis of officials’ and stakeholders’ public claims covered by the German news media. A dataset is compiled from news items containing the keywords ‘export surplus’ and ‘trade surplus’ at the German-language Reuters site. Reuters is an internationally oriented news agency targeted at business executives. It is a comprehensive source: notables events concerning the issue are reported here, including press releases and public statements (also those expressed in other media outlets). It excludes commentaries or opinion pieces. The studied timeframe is a nine-year period between 1 January 2010 and 31 December 2018.^2^ The period was chosen to include two important events when the surplus issue received heightened attention, the Eurozone crisis and the trade dispute with the Trump administration.

The database of 201 news items is further disaggregated, collecting stakeholder statements quoted by each news item. This yielded a database of 408 statements (an average of 2.02 per news item) which were hand-coded. There are two codes used—‘critical statements’ (CSs) mention the surplus in a negative context, ‘defensive statements’ (DSs) in a positive one.^3^ Stakeholders are categorised by the organisation they belong to. Note that only stakeholder statements (quotes) are recorded,’ descriptive reports (e.g. “last quarter, Germany’s export surplus reached a record high”) are not. Since quotes already display speakers’ positions, they help produce clear-cut categories. In the rare case of more neutral, “on the one hand … on the other hand” claims (e.g. Commission President Barroso or Minister for Economy Gabriel voiced cautious criticism while praising German competitiveness), *two* statements are recorded—one critical, one defensive.^4^ As a robustness check, the dataset is supplemented by an analysis of peak events through the coverage in two dailies of national reach (Frankfurter Allgemeine Zeitung, Süddeutsche Zeitung) and redtop daily Bild, as well as news broadcasts of German public television (ARD). Official policy documents are also reviewed.

A supplementary analysis turns to parliamentary debates in the Bundestag, using the online database of plenary protocols (DIP). In the studied period, a keyword search is used to identify 49 plenary debates (official government statements or Regierungserklärungen, planned debates, and parliamentary questions), where MPs discussed the issue of surpluses. A database of 101 remarks is compiled, which were also hand-coded.

The empirical strategy has limitations. While a qualitative technique with hand-coding allows deeper engagement with the complex frames, it introduces potential inaccuracies and biases. Moreover, the corpus of 201 news items and 49 parliamentary debates over a 9-year period shows that the overall frequency of discussing the surplus is rather low (unsurprisingly, given its technical nature). The discussion below also addresses what the ‘silences’ may imply for the analysis. The sample size suffices for the simple cross-tabulations required and for identifying broader patterns.

## Distributional costs mirrored in media narratives

**Fig. 3:**
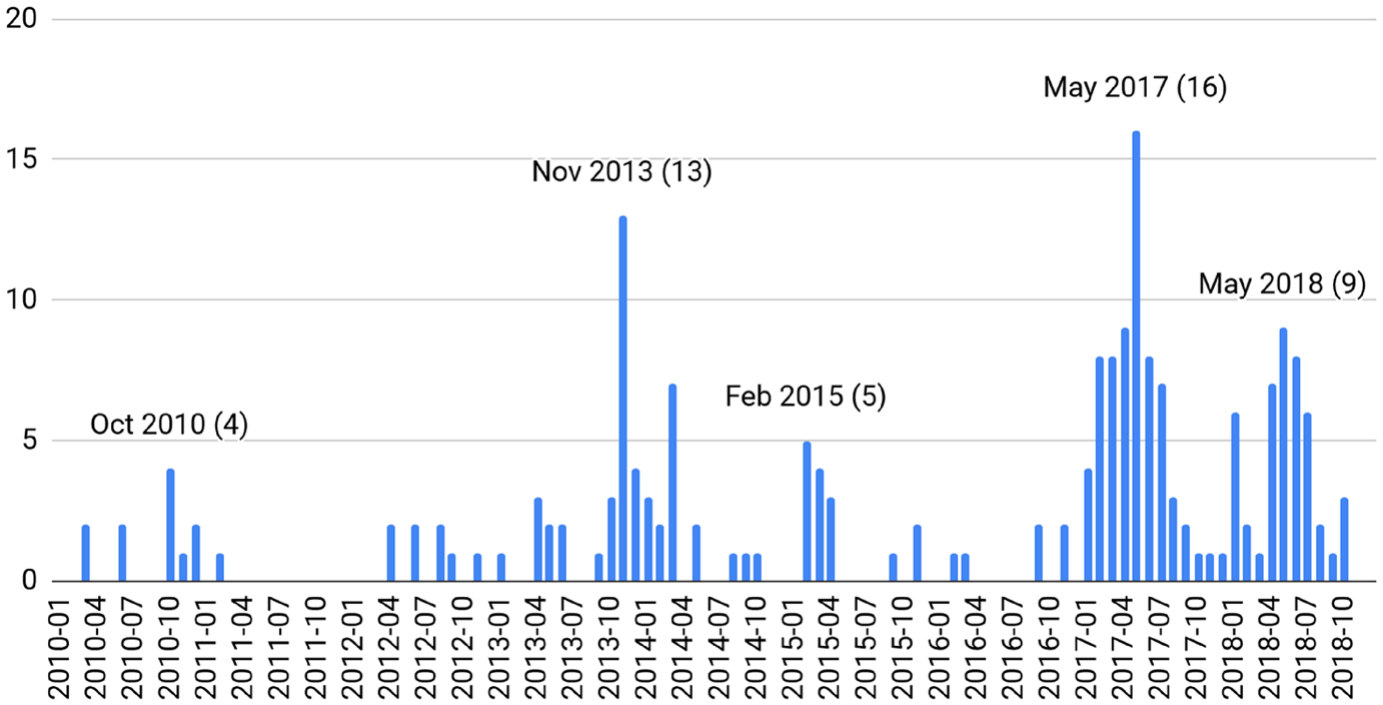
Monthly number of news items (data scraped from reuters.de)

What were the triggers of media attention, i.e. the events pushing the surplus issue onto the agenda? Plotting the monthly frequency of news items (Fig. 3), peaks indicate that it was outside criticism. The first peak was in October 2010, when Obama’s Treasury Secretary Geithner called out surplus countries and floated the idea of a corrective mechanism. In November 2013, two events occurred—the US Treasury expressed criticism in its official monitoring of trade practices and the European Commission announced its investigation into German imbalances.

In February 2015, the trigger was a German counteroffensive: turning the EU’s own criticism against them, Finance Minister Schäuble used the issue to criticise ECB policies weakening the euro. In early to mid-2017 and 2018, the Trump administration resumed Obama-era criticism in a harsher, threatening tone. This first finding already foreshadows the dynamic of the debate. The fact that attention turned to the issue due to outside criticism strengthens the perception that the surplus is someone else’s problem.

**Fig. 4:**
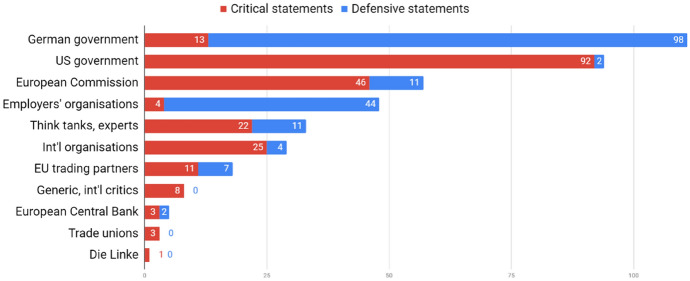
Number of critical and defensive statements by stakeholder category

Who are the main critics, and who are the defenders activated by criticism? Overwhelmingly, criticism originates from the outside. The US government (92 CSs), the European Commission (46 CSs), and international organisations (25 CSs) are the most prominent critics. The biggest defenders are the German government (98 DSs) and employers’ organisations (44 DSs). Trade unions only feature three times, voicing critical remarks. This piece of evidence supports the proposition that CA imbalances are framed as a conflict *between* countries, not *within* them. The issue arrives on the agenda from the outside, the government responds on the defensive. As surpluses pertain to ‘trade’ and ‘export’, the export industry’s voice is activated, while unions and left-wing parties remain silent, even though (as shown above) they are relevant stakeholders (Fig. 4).

The following section presents the content patterns (frames and arguments) revealed by critics’ and defenders’ remarks. The analysis succeeds chronologically, identifying six key stages.

### 1) Wage-dumping charges from Eurozone partners

The first highly publicised exchange in the period ensued in early 2010, as the euro crisis was recently underway. Officials representing Eurozone partners expressed their criticism with a focus on wages. French Finance Minister Christine Lagarde called Germany’s downward wage competition unsustainable. Luxemburg Prime Minister Jean-Claude Juncker accused Germany of having improved competitiveness through ‘wage and social dumping.’ German officials refused the charges, stressing that critics should rather follow Germany’s ‘no pain, no gain’ recipe to increase competitiveness and exports. Typical of these early narratives was the long report in German public television’s nightly news show, framing the problem as German companies prudently keeping wages low to safeguard employment and being scolded for their success. They asked: ‘should now the model student get worse just to get the class average right?’

### 2) The United States floating a penalty for surpluses

US Treasury Secretary Timothy Geithner criticised surplus countries’ contribution to external imbalances at a G20 summit and proposed an adjustment mechanism, maximising surpluses in 4% of GDP. The proposal triggered a fierce response from Finance Minister Schäuble:

The reason for Germany’s export success is not some kind of currency sleight-of-hand, but the increased competitiveness of our firms. The American growth model, on the other hand, is stuck in a deep crisis. (…) There are many reasons for the American economy’s problems. German export surpluses are not among them.

Schäuble makes a veiled reference to Americans’ frequent accusation of Chinese currency manipulation, dismissing it as a misguided assessment in the German case. Minister for Economy Rainer Brüderle (FDP) denounced Geithner’s “shocking relapse into planned economy thinking.”

### 3) Concerted calls for demand stimulus, timed for coalition talks

The peak in November 2013 consisted of two events—in a harshly critical report, the Obama administration condemned Germany's ‘anemic pace of domestic demand growth and dependence on exports’ (Department of Treasury 2013, p. 3), creating deflationary pressures globally. In 2 weeks, Commission President Barroso and Commissioner Rehn held a press conference announcing a probe into German export surplus (and weak domestic demand) within the macroeconomic imbalance procedure (MIP), a monitoring regime established in 2011. Again, ARD’s Tagesschau gave a typical summary of the German position, with an important nod to domestic costs:

The purpose of this investigation is above all a political one. It is difficult for other Europeans that they are tantalised for their problems while Germany is celebrating their successes. The Commission has to prove that they scrutinise everyone, including the good ones. They do not want and cannot do anything against Germany’s export strength. But their reminder that Germany can do more to revive domestic demand is not so easy to dismiss in view of broken motorway bridges and dilapidated school buildings.

It is important to note the *timing* of US and Commission criticism. This was the immediate aftermath of the 2013 German federal elections, as Union and SPD were informal coalition talks. It is plausible that outside pressure was increased to influence outcomes in a susceptible moment when a range of questions was opened for negotiation.

### 4) Incremental change: minimum wage and investments

In November 2013, a new government was formed, and centre-right FDP was substituted by centre-left SPD as junior partner—positions and policies changed. The most important measure from the aggregate demand point of view was the introduction of the statutory minimum wage of EUR 8.50, effective from January 2015.

Early 2014, Süddeutsche Zeitung leaked a government position paper acknowledging the persistent surplus as a problem for Eurozone stability. The brief for Minister for Economy Sigmar Gabriel concluded that “reasons are complex, one important driver is the weakness of investments.” Later that year, Gabriel appointed an Expert Commission led by Economist Marcel Fratzscher to propose steps to boost investments. However, careful rhetoric foreshadowed careful outcomes: the government stuck to balanced budget policies, undermining any investment strategy. As investments rose somewhat, officials started emphasising that it was ‘highest in years’ and all capacities were utilised.

### 5) Shifting strategies: ‘the best defense is a good offense’

From 2015 onwards, there is a visible discursive shift—mirroring a shift in officials’ strategy. This was the time the European Central Bank started its asset purchase program amidst fierce German resistance. Exceptionally, it was Finance Minister Schäuble bringing up the surplus issue on multiple occasions to voice dissent against low rates and quantitative easing (QE). As he explained: “The bond purchases will lead to a bigger German surplus. It is quite ironic that those who argue for QE then criticise Germany.”

### 6) Threats of the Trump administration

Donald Trump took office with a highly politicised position on bilateral trade deficits. Although his focus was China, shortly before inauguration, he put Germany in his crosshairs too. In an interview, he threatened German carmakers with a 35% tariff. Minister Gabriel fired back that the US should ‘build better cars.’ Shortly thereafter, Economic Advisor Peter Navarro accused Germany of currency manipulation. From January to May 2017 (as media attention peaked again), German government members responded to intensifying threats with every rhetorical tool of previous years (see Table 2).

**Table 2:** German responses to the Trump tariff threats

	Quote	Strategy
Sigmar Gabriel, Jan 16, 2017	rather than trying to penalise German carmakers, the US should instead respond by building better and more desirable cars	Pointing to market forces
Wolfgang Schäuble, Feb 5, 2017	Also in Washington, it will soon sink in that European monetary policy is not made by the German Government, but by the European Central Bank. And they will also realise that the German Finance Minister is not exactly a fervent fan of this monetary policy	Arena-shifting (plus ECB criticism)
Angela Merkel, March 10, 2017	Trade policy is a responsibility of the European Union	Arena-shifting
Brigitte Zypries, March 12, 2017	I believe the governor of South Carolina has no interest in BMW taking away jobs or reducing investment there	Appealing to trading partners’ benefits
Brigitte Zypries, April 19, 2017	Germany's economy is competitive and strong. No one has to apologise for the fact that our high-quality machines and equipment are in demand from abroad	Pre-eminence of the German model
Wolfgang Schäuble, April 20, 2017	There are no sensible measures to reduce Germany's current account surplus, nor do we need active economic policy intervention to achieve this	Stressing limited policy discretion

## Domestic contestation in the German Bundestag

The other part of the analysis examines how the issue filtered into the parliamentary arena. In the studied period, keyword searches identified 49 plenary debates discussing CA surpluses. A database of 101 remarks is compiled, which are hand-coded as critical (62) and defensive (39).

**Fig. 5:**
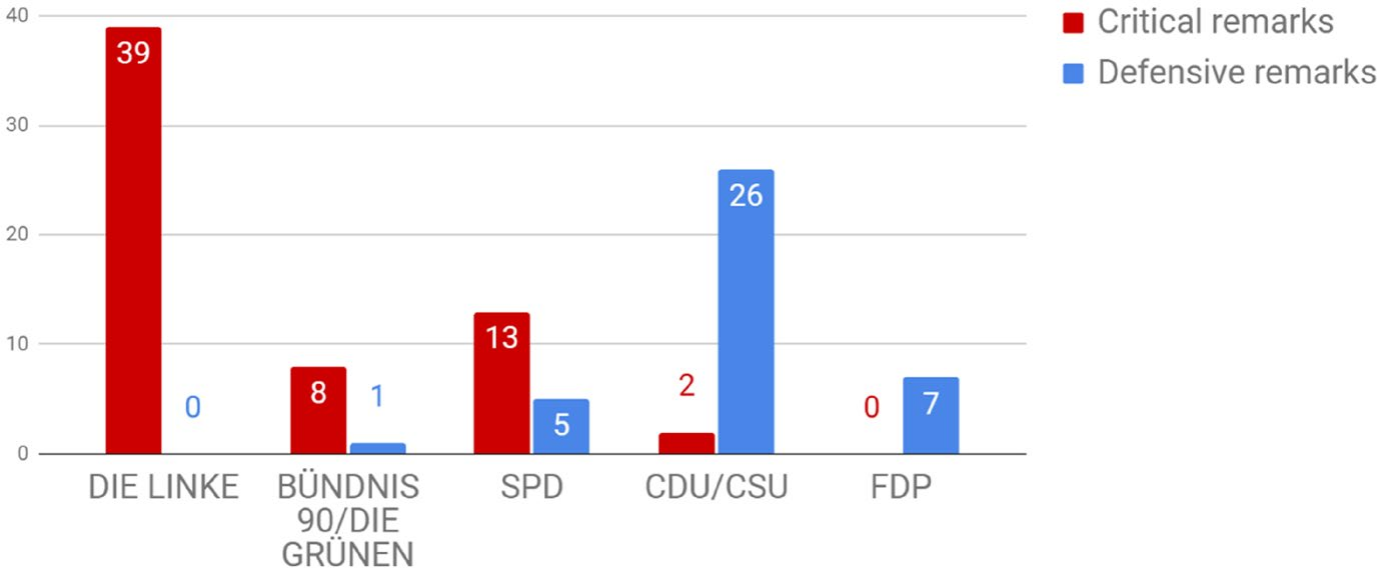
Number of critical and defensive statements by party

Plotting remarks by party (Fig. 5), The Left (Die Linke) stands out as the most, and arguably the only active critic. Also critical is the Green Party (Bündnis 90/Die Grünen), although with little attention to the topic. Social Democrats (SPD) are divided in the orientation of their remarks—explained by their move from opposition (2009–2013) to government (2013–2017). In the first electoral period, SPD MPs’ remarks were critical (10–0), in the second one, mostly defensive (3–5). Most defenses are coming from centre-right Christian Democratic Union (CDU), senior partner in both governments. While they were in parliament (2009–2013), liberal Free Democratic Party (FDP) was also a staunch defender of surpluses. Ideological positions of the parties largely explain their positions.

The Left and to a lesser extent, the Greens frame the surplus as a problem for the German domestic economy—with frequent references to suppressed wages, also criticising that surpluses contribute to deficit countries’ indebtedness. The Left also uses frames like stability (accusing the government of violating Germany’s Stability Act) and pro-Europeanism. There are multiple MPs expressing solidarity with austerity-stricken periphery countries. This is consistent with the findings of Kinski (2018) who identified a pattern of ‘Eurosceptic Europeanisation’: Eurosceptic (left-wing) MPs claiming to represent European citizens as well.

Representatives of FDP and CDU/CSU, and while in government, also SPD often argued that the surplus is a market outcome, a result of German firms’ hard work and competitiveness. FDP politicians repeatedly stated that ‘anyone who demands the reduction of the German export surplus in Germany or abroad wants to reduce the number of jobs,’ conflating the surplus with exports (reducing a surplus, a *difference*, could as well mean increasing imports).

Arguments focused on market forces are often paired with allegations that those who wish to correct surpluses are against the principle of free trade, do not believe in competition, and want to succeed without hard work or by cheating. Football metaphors are numerous: a CDU MP compared rebalancing to “German national football teams sending off (captains) Birgit Prinz and Philipp Lahm to help opponents.” MPs also emphasise that they do not accept a sort of ‘downward balancing’ in Europe, as ‘weakening the strong will not strengthen the weak.’

Problematisation of the issue often comes from abroad. Out of 101 remarks, over one-third, 34 explicitly mention critics (29 in critical, 5 in defensive context). Each contestation episode outlined by the media analysis is mentioned at least once by parliamentarians. Criticism from Brussels is referenced the most: critical MPs cite that Germany violates EU regulations and use European partners’ condemnation to underscore their point. Defenders (14) refer to the Commission’s *positive* judgment (emphasising that no excessive imbalances were ever found) and criticise European authorities for bringing up the surplus at all.

## Discussion of results

Not only defenders, but also most critics stay in the competitiveness (expenditure switching) narrative, in line with expectations. Both Europeans’ unfair wage-dumping critiques (Stage 1) and Americans’ nominal exchange rate manipulation charges (Stage 6) are competitiveness-based. Critics often accuse Germany of beggar-thy-neighbour exchange rate devaluation through an undervalued euro or unfair advantages through ‘wage dumping’ (note that low wages are not framed as a weak demand problem, but as a competitive edge). However, as shown above, German imbalances are not (certainly not *only*) rooted in relative prices, but the domestic *level* of spending which could be adjusted by *expenditure changing*. Nominal exchange rate movements explain the size of the surplus to a limited extent, as it was already excessive at stronger USD/EUR rates. Critics erroneously sticking to the expenditure switching framework let German policymakers off the hook: they can easily rebut criticisms, since exchange rates and trade are European-level prerogatives, and Germany does not advocate for a weak euro—quite the contrary, as the Schäuble versus ECB debate (Stage 5) shows (see also: Brunnermeier et al. 2016, p. 74).

Although most critics and defenders stay in the competitiveness framework, there are notable exceptions—Obama-era US critics, and partly also the messaging from the European Commission (Stage 3) identified weak domestic spending as the key problem. Authorities seemed more open to these critics, taking some (timid) steps to boost aggregate demand, largely at the urging of SPD (Stage 4). Importantly, these incremental steps were not linked to the surplus issue in public communications. The critical position paper was a leak, and SPD politicians endorsed it only half-heartedly, albeit following up on its policy recommendations. This implies the willingness to do something about the imbalance problem without openly linking interventions to it, being wary of its profound counter-intuitiveness as a message. Coalition dynamics on the one hand and public opinion on the other restrain the SPD’s deviation from the hegemonic narrative. Proactive criticism of the surplus remained a fringe position, which also explains the ‘silences’ in the datasets—shown, among others, by the little attention to the topic by the left-leaning Green Party, or the fact that media attention is triggered almost exclusively by foreigners.

Patterns follow partisan lines. Those with a more right-wing agenda are the fiercest defenders of surpluses: stressing that they are signs of competitiveness reached by painful reforms (that others are free to follow); and since the driver is market demand for products ‘Made in Germany,’ the government has limited tools to reduce it—invoking a depoliticisation or ‘No Alternative’ logic (Watson and Hay 2003). They also discredit critics by pointing to their economy success compared to crisis-stricken competitors. Plausibly because of former President Trump’s unpopularity, defensive reactions to *his* criticism were particularly enthusiastic, across the political aisle.

The harsher the criticism, the stronger the ‘us versus them’ or identitarian framing. German successes on export markets are often narrated as a source of national pride (akin to football triumphs) and export strength is part of ‘who we are.’ ‘We are an export nation’ (‘Wir sind Exportnation’) and ‘we are export world champions’ are recurring tropes invoked in response to outside criticism.

## Deficiencies in international and European economic governance

To address how Germany gets away with its overreliance on trading partners, this final section turns to the global and European levels.

The fundamental institutional deficiencies of the global economic order were already foreshadowed by the famous debate between John Maynard Keynes and Harry Dexter White in Bretton Woods (Eichengreen and Temin 2010). Keynes advocated for a so-called ‘clearing union’, an institutionalised corrective mechanism to curtail surpluses as well as deficits. White, representing the US (ironically, the main proponent of such a mechanism today), had a vested interest against it, as they were running large and consistent surpluses vis-à-vis Britain’s deficits. White had the upper hand, and a clearing union never came to fruition. Although the idea of surplus country adjustment was revived in the wake of the crisis (Jones 2009), there are limited means to achieve it. As opposed to deficits, where changes in investor sentiment and sudden stops of capital inflows can force a sharp and painful adjustment, surplus countries cannot be forced to adjust by market pressures. There is an inherent asymmetry here: excessive borrowing can be stopped by the lenders, but excessive lending (or saving) cannot.

There is one market manifestation of Keynes’ plan for a corrective mechanism: negative interest rates, which work as a de facto fine on excess savings. Interest rates are steered by the supply and demand of financial assets (savings vehicles)—and they are in part so low because of Germany’s excessive saving desire. While German officials warn against meddling in market outcomes when it comes to surpluses, they see interest rates differently. Rates are understood to be determined by ECB discretion (even though economists point to an ‘equilibrium market rate,’ a market price the ECB does not set but follows). German authorities, even joined by the Constitutional Court, vehemently criticise the ECB.^5^ They denounce penalties on excess savings—again, contesting the profound moral counter-intuitiveness of ‘too much saving.’

European coordination, although taking steps in the direction, proved unable to constrain Germany. An important episode was the MIP—a surveillance tool with a corrective arm, established in 2011. One of the MIP’s novelties was that it monitors surpluses as well as deficits, although with different weights: thresholds are 3% for deficits, 6% for surpluses (DG ECFIN, 2012, p. 8). Beyond the asymmetry (dubbed ‘intelligent symmetry’) of cut-off values, Darvas and Leandro (2015) show MIP monitoring gradually abandoned recommendations regarding surpluses: by 2015, the aim of symmetric adjustment disappeared altogether. The overall credibility of the MIP’s surplus rule is weak. The excessive imbalance procedure was never launched, and Germany pushed through a declaration stating that large and sustained surpluses are not as problematic as deficits, so they do not warrant sanctions (Council of the EU, 2011, p. 9).

Not only does the EU have a flawed institutional framework to correct imbalances (Moschella 2014), European integration can also exacerbate imbalances (Johnston and Regan 2018). The channel identified by the above analysis is arena-shifting (Flinders and Buller 2006). As trade policy is delegated to the EU level, German officials usually refer critics to Brussels (and Frankfurt). The EU is the world’s largest trading bloc, wielding immense leverage over trading partners (bigger than Germany alone), making it difficult to strong-arm them into concessions. Monetary policy is also delegated. German policymakers use this to criticise ECB’s monetary stimulus, claiming that the surplus is driven up by QE—even though it is implausible. While QE does indeed weaken the euro, monetary stimulus has a positive impact on domestic demand through the expenditure changing channel, raising the level of spending.

The German side rarely acknowledges that beyond explicitly trade-linked policies like nominal exchange rates or tariffs, domestic macroeconomic policies have an impact on trade, and those policies are the German government’s prerogative to change.

## Conclusion

The analysis showed how Germany’s excessive current account imbalances—although mirroring domestic distortions like rising inequality, in-job poverty, and underinvestment—are portrayed as a distributional tension *between* countries not *within* Germany. Instead of discussing the underlying domestic demand problem at the root of excessive imbalances, the debate is thus side-tracked into profoundly counterintuitive bickering about the German economy being ‘too competitive’ and ‘exporting too much.’ As evidenced above, surpluses are almost exclusively problematised by outside actors—mainly US administrations and the European Commission. This criticism, in turn, activates the German government and representatives of the exporting industry, who respond with a fierce defense of ‘the German model.’ The national interest is portrayed as monolithic and associated with the interests of export sector producer groups, as opposed to consumers or domestically-oriented sectors. The logic of this ‘us versus them’ debate leaves little space for actors representing the domestic losers of surpluses (like trade unions or left-wing parties), their voice remains muted. This contributes to the blunted domestic pushback against the policies upholding imbalances and may help explain the puzzling absence of German rebalancing.

The story of a proud export nation with record-breaking surpluses akin to football triumphs just cannot be squared with harsh criticism from problem children like the US or Eurozone partners. Considering the passionate defenses they trigger, attacks often seem to backfire, and rather reinforce the narrative of a successful German economy, the envy of the world. Against this backdrop, domestic critics have a hard time expressing more overarching, programmatic reform proposals or raise awareness to systematic problems, contributing to the stance Bremer and McDaniel (2019) call ‘the ideational foundations of social democratic austerity.’ An important avenue of further research could explore how this discursive bias against left-wing voices like trade unions may interact with and feed into structural causes of the erosion of these actors’ power (e.g. Hassel 2014). The only consistently and proactively critical voice is small, far-left Die, Linke—bizarrely sharing a platform with the Obama administration or conservative-affiliated Christine Lagarde in the surplus issue.

As a further original contribution, the analysis shed light on the limits of European coordination to rein in surpluses. The EU took timid steps towards the monitoring and curbing of German imbalances—but European integration also has adverse impacts that end up exacerbating them (Johnston and Regan 2018). Integration opens an opportunity for an arena-shifting strategy: as the debate remains fixated on traditional levers of trade policy like exchange rates and tariffs (as opposed to domestic macroeconomic policies), the German side can refuse direct responsibility, stress their limited policy discretion, and emphasise that both monetary and trade policy are delegated to the European-level.

These insights are tied to a wider discussion about the destabilising effects of domestic distortions in an open, globalised economy, and how disregarding domestic-level issues like social inequality or underinvestment may thwart the correction of global imbalances (Klein and Pettis 2020). Outside critics have limited ways to rectify the weakness of household consumption or public investment in surplus countries—even though in the context of weak demand and near-zero interest rates, they are directly hurt by it. The key is held by domestic residents, whose purchasing power is undermined and who suffer from an erosion of public goods like infrastructure, but who are rarely part of the imbalances discussion. Although trade in the twenty first century is organised in value chains spanning multiple continents and is increasingly decoupled from nation-states, it is narrated as a noisy rivalry *between* nations, drowning out dissatisfied voices *within* them.

## Data Availability

A replication file with the database of news items and stakeholder statements; as well as all data and calculations featured in this article is openly available at the Harvard Dataverse under https://doi.org/10.7910/DVN/SRF1BG.

## Footnotes

1 “German business calls for end to new borrowing ban” Sep 24, (2019), Financial Times; “BDI-Chef Kempf hinterfragt deutschen Handelsüberschuss” Sep 14, 2017, Reuters.

2 The 2010–2018 period roughly corresponds to two German electoral periods between Oct 27, 2009 and Oct 24, 2017. The starting point was chosen due to data availability: articles were accessible from Jan 1, 2010. The year 2018 was included to cover an important episode (the Trump trade war).

3 The datasets are openly available on the Harvard Dataverse.

4 Codes refer to the substance of statements, *tone* within the two categories does differ—politicians use stronger language than more neutral experts.

5 “Germany’s ECB critics toast courtroom success” Financial Times, May 8, (2020).
